# Recent Advances in C–C and C–N Bond Forming Reactions Catalysed by Polystyrene-Supported Copper Complexes

**DOI:** 10.3390/molecules22060865

**Published:** 2017-05-24

**Authors:** Pavel Drabina, Jan Svoboda, Miloš Sedlák

**Affiliations:** Institute of Organic Chemistry and Technology, Faculty of Chemical Technology, University of Pardubice, Studentská 573, 532 10 Pardubice, Czech Republic; pavel.drabina@upce.cz (P.D.); jan.svoboda@upce.cz (J.S.)

**Keywords:** copper complexes, C–C bond forming reaction, C–N bond forming reaction, polystyrene, supported catalyst, recyclable catalyst

## Abstract

This present mini-review covers recently published results on Cu(I) and Cu(II) complexes immobilized on polystyrene carriers, which are used as heterogeneous, eco-friendly reusable catalysts applied for carbon–carbon and carbon–nitrogen forming reactions. Recent advances and trends in this area are demonstrated in the examples of oxidative homocoupling of terminal alkynes, the synthesis of propargylamines, nitroaldolization reactions, azide alkyne cycloaddition, *N*-arylation of nitrogen containing compounds, aza-Michael additions, asymmetric Friedel–Crafts reactions, asymmetric Mukaiyama aldol reactions, and asymmetric 1,3-dipolar cycloaddition of azomethine ylides. The type of polystyrene matrix used for the immobilization of complexes is discussed in this paper, and particularly, the efficiency of the catalysts from the point of view of the overall reaction yield, and possible enantioselectivity and potential reusing, is reviewed.

## 1. Introduction

Preservation of the current standard of living for the human population is not further possible without the development of ecologically sustainable chemical processes and technologies [1,2]. One of the methods for contributing to responsible handling with raw materials and energy resources is the utilization of recyclable catalysts [1,2,3,4,5]. The strategy of how to obtain recyclable catalysts belongs the immobilization of known homogeneous catalysts to the solid carriers [3,4,5]. Organic polymers, especially commercially available cross-linked pearl-like copolymers of styrene e.g., Merrifield resin™ [6] and JandaJel resin™ [7] belong among the most relevant carriers. The remarkable advantage of polymer-supported catalysts lies in their simple separation and reusability, their low price, and also in the possibility of their usage in selective continuous-flow procedures [5,7]. The immobilization of the catalyst can be performed by the reaction of ligands or catalysts with suitably chemically activated polystyrene (post-modification strategy) [7]. Another possibility is the grafting of the ligand or catalyst containing a double bond from the reaction, with monomers generating the scaffold of the polymeric carrier (copolymerization strategy) [7]. The convenience of one or other strategies cannot be generally stated—it depends on many factors for each concrete case. Nowadays, there are many examples in chemical databases of recyclable catalysts based on complex compounds immobilized on different polymers. This review is focused on the area of highly efficient recyclable catalysts based on Cu(I) and Cu(II) complexes immobilized on polystyrene carriers. The aim of this review was to summarize, discuss and evaluate recently published results of catalysts, which were designed particularly for reactions, where new carbon–carbon respective carbon–nitrogen bonds are formed.

## 2. Carbon–Carbon Forming Reactions

### 2.1. Oxidative Homocoupling of Terminal Alkynes

**Scheme 1:**
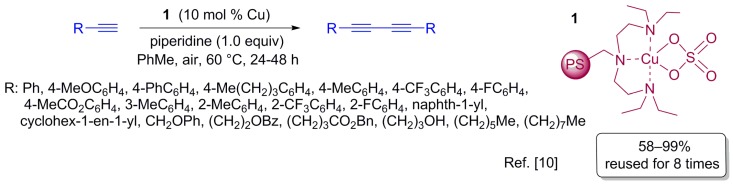
Oxidative homocoupling of terminal alkynes catalysed by polymeric complex **1**.

Carbon–carbon bond forming reactions are essential tools for building the carbon skeleton of organic compounds, and therefore they represent an elemental content of organic synthesis. Copper-catalysed oxidative homocoupling of terminal alkynes known also as Glaser coupling [8] introduce a suitable method for the preparation of 1,3-diynes, which are needful basic building blocks in the synthesis of many natural and pharmaceutical products, as well as some advanced functional materials [9]. A successful application of the heterogeneous catalyst intended for this reaction can be documented on the example of recyclable catalyst **1**, which is based on the complex consisting of *N*,*N*,*N*′,*N*′-tetraethyldiethylenetriamine and CuSO_4_, anchored on polystyrene cross-linked with 1% divinylbenzene (DVB) (Scheme 1) [10].

The efficiency of catalyst **1** was confirmed from the syntheses of 20 substituted symmetrical 1,3-diynes, where high yields of isolated products were achieved (58–99%). During the reuse of catalyst **1**, release of small amount of Cu(II) ions was observed, however this did not affect significantly the yield of 1,4-diphenylbutane-1,3-diyne, even after nine cycles [10]. Another recently published successful heterogeneous catalyst [11] for Glaser coupling was based on the Cu(II) complex of copolymer *N*-(1,10-phenanthroline-5-yl)acrylamide and DVB. This catalyst was also used for Huisgen 1,3-dipolar cycloaddition [11]. For oxidative C–C homocoupling of acetylenes, a heterogeneous catalyst based on copper nanoparticles (3–9 nm), stabilized onto nitro functionalized polystyrene resin, was prepared. This catalyst was tested in the synthesis of seven 1,4-disubstituted-1,3-diynes (92–95%) [12].

### 2.2. Sonogashira Cross-Coupling Reaction

**Scheme 2:**
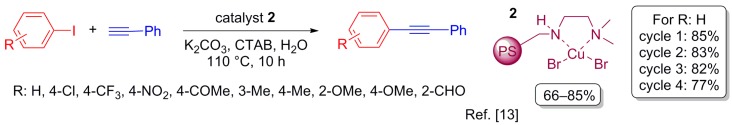
Sonogashira cross-coupling reaction catalysed by catalyst **2**.

Sonogashira cross-coupling of ten substituted iodobenzenes with phenylacetylene catalysed by polystyrene-supported Cu(II) complex **2** was described by Mandapati et al. [13] (Scheme 2). Heterogeneous catalyst **2** was prepared by the reaction of commercial chloromethylated polystyrene (5.5% DVB) with *N*,*N*-dimethylethylenediamine and subsequent complexing with CuBr_2_. Cross-coupling reactions proceeded in aqueous media in the presence of K_2_CO_3_ and cetyltrimethylammonium bromide (CTAB) at 110 °C for 10 h. The yields of substituted acetylenes ranged between 66–85%. Reusability of the catalyst was tested for the reaction of iodobenzene with phenylacetylene (Cycle 1: 85%; Cycle 2: 83%; Cycle 3: 82%; Cycle 4: 77%) (Scheme 2).

The main advantage of this system lies in easy preparation of the catalyst from inexpensive and commercially available starting materials. Moreover, a significant benefit is the possibility of reusing of the catalyst, and the eco-friendly performance of the reaction in aqueous media [13].

### 2.3. Carbene Transfer Reaction

**Scheme 3:**
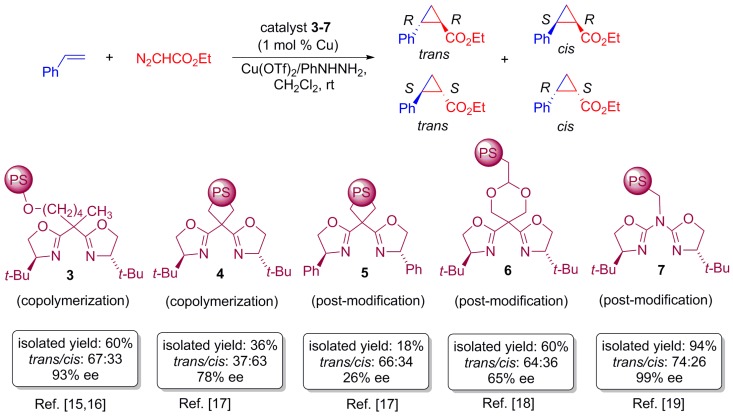
Cyclopropanation catalysed by different polystyrene-supported complexes **3**–**7**.

Reactions where carbenes were transferred onto unsaturated substrates (alkenes, alkynes, arenes, imines, aldehydes etc.), have provided a number of useful products [14,15,16,17,18,19]. The mentioned carbenes can be generated in situ from diazo compounds by the application of transition metal-based catalysis [14]. The most widespread example of such reaction implemented at large scale is copper-catalysed cyclopropanation of substituted alkenes with alkyl diazoacetates. Therefore, many homogeneous as well as heterogeneous catalytic systems have been developed for this reaction [14,15,16,17,18,19,20]. In the case of Cu(I) complexes, bisoxazoline ligands [15,16,17,18] and azabisoxazoline ligands [19] were used, anchored on pearl-like polystyrenes. For the immobilization of ligands either a copolymerization strategy (catalysts **3** and **4**) or post-modification strategy (catalysts **5**–**7**) was utilized. The catalysts were generated in situ by the coordination of copper(II) triflate and consequent reduction with phenylhydrazine (Scheme 3) [15,16,17,18,19,20].

Catalyst **3** was prepared by copolymerization of corresponding bisoxazoline-bearing styrene (4%) with styrene (42%) and DVB (54%), which gave a highly cross-linked polymer having a compact structure (<5 m^2^ g^−1^) [15,16]. Cyclopropanation catalysed by **3** was tested on the reaction of ethyl diazoacetate with styrene (60%, *trans*/*cis*: 67/33, 93% enantioselective excess (ee)) (Scheme 3); with 1,1-diphenylethene (97%, 98% ee) and with 2-methylpropene (92%, 97% ee) [16]. Catalyst **3** was fivefold reused in the reaction of styrene with ethyl diazoacetate without any significant decrease in yield and enantioselectivity. Modification in the preparation of catalyst **3** by performing of copolymerization in different ratios of bisoxazoline-bearing styrene (6%) with styrene (92%) and DVB (2%) did not affect either the isolated yield (67%), or the diastereoselectivity (67/33) and enantioselectivity of cyclopropanation (92% ee) [16]. Likewise, a change of strategy of preparation for catalyst **3** (post-modification of Merrifield resin™) did not lead to any significant impact on the catalyst′s efficiency [16]. Poor enantioselectivity found for catalysts **4** (36%, *trans*/*cis*: 37/63, 78% ee) [17] and **5** (18%, *trans*/*cis*: 66/34, 26% ee) [17], and **6** (60%, *trans*/*cis*: 64/36, 65% ee) [18], was explained by lower binding affinity towards copper, caused by the change of geometry of the ligand as a consequence of the replacement of the *gem*-dimethyl substitution box-bridge with sterically large groups [19]. A notable improvement in binding affinity towards copper was achieved by using of azabisoxazoline ligand; moreover, it also led to an increase of the catalyst **7**’s efficiency (94%, *trans*/*cis*: 74/26, 99% ee) [19]. An improvement in enantioselectivity (79% ee, *cis*)—explained by the effect of the “chiral-cavity” of the rigid polymer—was later achieved for the highly-crosslinked catalyst **4**, which was prepared according to the copolymerization strategy [20]. Even in this case, the influence of post-modification strategy or copolymerization strategy was not significant. On the other hand, testing confirmed that the Merrifield resin™ used in catalyst **7** was a much better support than TentaGel™ (35%, *trans*/*cis*: 64/34, 47% ee) [19].

**Scheme 4:**
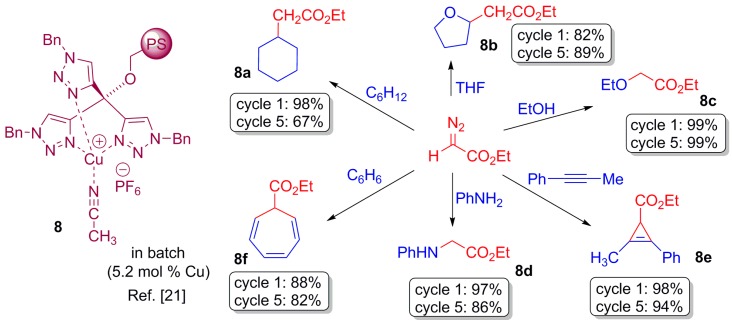
Carbene transfer reactions catalysed by polymeric complex **8**.

The next reusable catalyst **8** was prepared by coordination of [Cu(MeCN)_4_][PF_6_] on tris(triazolyl)methane anchored on commercial Merrifield resin™ (Scheme 4) [20]. Reactions of catalyst **8** with ethyl diazoacetate led to formation of carbene (:CHCO_2_Et), which was used for six reactions with following substrates: cyclohexane (C–H insertion), tetrahydrofuran (THF) (C–H insertion), ethanol (O–H insertion), aniline (N–H insertion), phenylacetylene (cyclopropenation), and benzene (cyclopropanation/ring expansion). In the case of the usage of benzene as substrate, this was the first representative of the Büchner reaction performed under the conditions of heterogeneous catalysis (Scheme 4) [21].

The reactions were performed both in batch and, for the first time, also in continuous flow. In the first variant, the reaction proceeded in a Schlenk flask under an inert atmosphere. Minor by-products were detected—only diethyl fumarate and diethyl maleate. Products of the Cu-catalysed carbene transfer addition or insert reaction **8a**–**f** were obtained with high yields (**8a**: 98%; **8b**: 82%; **8c**: 99%; **8d**: 97%; **8e**: 98%; **8f**: 88%). Moreover, the yields were still satisfactory during experiments with catalyst recycling and its reuse; e.g., after the fifth cycle the results were as follows—**8b**: 89%; **8c**: 99%; **8d**: 86%; **8e**: 94%; **8f**: 82%. A significant decrease of yield was observed with product **8a** (67%), which was explained by the lower reactivity of cyclohexane during insertion into the C–H bond [21]. In the continuous flow arrangement, a vertically mounted and fritted low-pressure Omnifit column loaded with catalyst **8** was used. Very stable flow was afforded after two hours with the following results—**8a**: 0.94 mmol; 41%; 4 mmol_product_·mmol_Cu_^−1^ h^−1^; **8b**: 2.53 mmol; 56%; 10.8 mmol_product_·mmol_Cu_^−1^ h^−1^; **8d**: 3.91 mmol; 89%; 16.7 mmol_product_·mmol_Cu_^−1^ h^−1^; **8e**: 0.54 mmol, 70%; 2.3 mmol_product_·mmol_Cu_^−1^ h^−1^; **8c**: (after 48 h) 12.5 g; 17.1 mmol_product_·mmol_Cu_^−1^ h^−1^; turnover number (TON) = 820. From the given results, catalyst **8** represents a highly efficient catalytic system for carbene transfer reactions to various types of substrates both in batch and in continuous flow [21].

### 2.4. Addition of Alkynes on Double Bond Carbon–Nitrogen

**Scheme 5:**
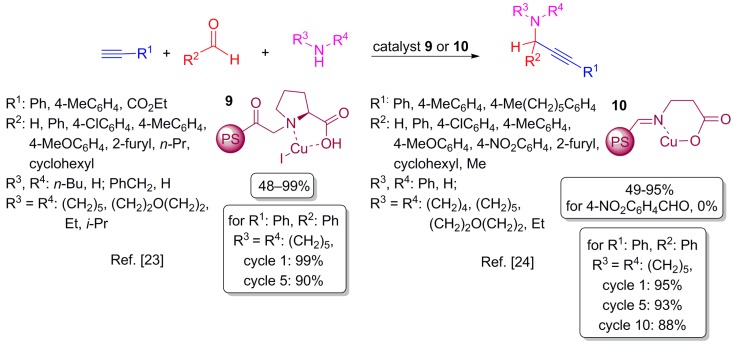
Addition of terminal alkynes to imines catalysed by catalysts **9** and **10**.

**Scheme 6:**
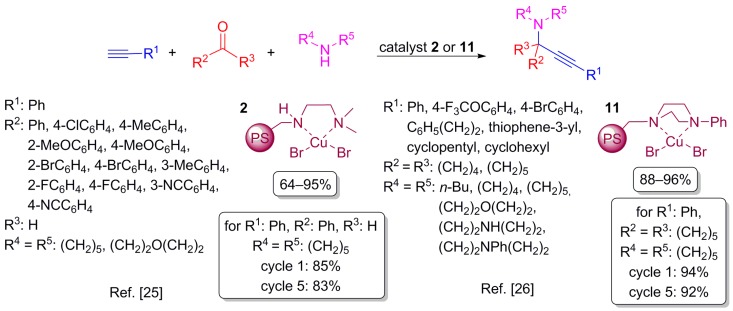
Addition of terminal alkynes to imines catalysed by catalysts **2** and **11**.

Propargylamines—which represent important intermediates for the synthesis of significant pharmaceutical polyfunctional substances—can be synthesized by the addition of alkynes on the imine bond (Scheme 5 and Scheme 6) [22,23,24,25,26]. Propargylamines had been traditionally prepared by nucleophilic addition of magnesium or lithium acetylides on imines or their derivatives [22]. The disadvantage of such synthesis consists in the necessity of use of stoichiometric amounts of acetylides under very strict reaction conditions and absolute moisture exclusion. Simplification of this reaction brought the utilization of transition metal complexes—especially Cu(I) complexes [22]. Unfortunately, the isolation of these homogeneous complexes from the reaction mixture as well as their recycling is unfeasible. Further facilitation started with the application of Cu(II) complexes immobilized on pearl-like polystyrene and with the reaction performed as a one-pot multi-component reaction [23,24,25,26]. Catalyst **9** was prepared by the reaction of l-proline with chloroacetylated pearl-like polystyrene and subsequent complexation with CuI (Scheme 5) [23]. The reaction of all three components (alkyne/aldehyde/amine: 1/2/1.25; 5 mol % Cu) catalysed by **9** proceeded under laboratory conditions [Dimethyl sulfoxide (DMSO), 2 h or 12 h at room temperature] with high yields of isolated propargylamines (48–99%). Formaldehyde and aliphatic aldehydes provided higher yields (78–99%) than aromatic aldehydes (e.g., Ph-C≡CH/4-MeO-C_6_H_4_CH=O/piperidine: 48%). For the reaction of phenylacetylene, formaldehyde and piperidine catalysed by catalyst **9**, several solvents were tested: water (0%), methanol (25%), toluene (30%), THF (42%), MeCN (87%), DMSO (99%). For the same reaction in DMSO, the recovery and reuse of catalyst **9** was tested, when only slight decrease in yields for each cycle was observed: 99%, 99%, 98%, 95%, 90%, 90% (Scheme 5) [23]. Corresponding polymer complexes with CuBr, CuCl and CuSO_4_ were also studied; however, they were less efficient [23].

Catalyst **10** was prepared by the reaction of β-alanine with polystyrene containing the –CH=O group and by subsequent complexation with CuI (Scheme 5) [24]. The reaction catalysed by catalyst **10** was performed under reflux in water (6–16 h) with the following ratio of educts: alkyne/aldehyde/amine: 1/1/1.2 (2 mol % Cu), while the obtained yields of isolated propargylamines ranged between 48–99%. For the reaction of phenylacetylene, benzaldehyde and piperidine catalysed by **10** also different solvents were tested: dichloromethane (DCM) (15%), toluene (58%), MeCN (62%), THF (68%), water (95%). Furthermore, the recovery and reuse of catalyst **10** was studied for the same reaction in water, where a slight decrease in yields was observed: Cycle 1: 95%; Cycle 5: 93%; Cycle 10: 88%. Besides, the authors monitored the copper content in catalyst **10**, which remained unchanged in all of the cycles (Scheme 5) [24].

Catalyst **2** (Scheme 2) was also used by the same authors [13] for the synthesis of substituted propargylamines (Scheme 6) [25]. The reaction of all three components (alkyne/aldehyde/amine: 1.1/1/1.1; 1 mol % Cu) catalysed by catalyst **2** proceeded in toluene (6 h, 110 °C) with yields of isolated propargylamines in the range of 64–95% (Scheme 6). Also in this case, different solvents were tested for the reaction of phenylacetylene, benzaldehyde and piperidine catalysed by catalyst **2**: water (trace), methanol (trace), ethanol (trace), THF (22%), MeCN (36%), dimethylformamide (DMF) (48%), dichloroethane (DCE) (46%), DMSO (50%) and toluene (85%). The recovery and reusing of catalyst **2** was studied for the same reaction, again with only slight decrease in yields: Cycle 1: 85%; Cycle 5: 83% (Scheme 6) [25]. Catalyst **11** was prepared by the reaction of commercial chloromethylated polystyrene with *N*-phenyl piperazine and subsequent complexation with CuBr_2_ (Scheme 6) [26]. The reaction of all three components (alkyne/ketone/amine: 1/1/1; 0.7 mol % Cu) catalysed by **11** was performed under solvent free conditions (6 h, 110 °C) with the yields of isolated propargylamines in the range of 88–95%. The recovery and reusing of catalyst **11** was tested for the reaction of phenylacetylene, cyclohexanone and piperidine, while only negligible decrease in yields was also observed: Cycle 1: 94%; Cycle 5: 92% (Scheme 6) [26].

From the presented results, the prepared heterogeneous catalysts **2**, **9**–**11** represent highly efficient catalytic systems. Particularly, catalyst **11** showed itself to be an eco-friendly variant that allowed the synthesis of propargylamines in aqueous media. From the chemical structure of catalyst **9**, it contains a defined stereogenic centre. However, the eventual optical purity of chiral propargylamines prepared with the use of this catalyst was not discussed in the cited paper [23]. Elsewhere, e.g., in [27,28,29,30], several enantioselective homogeneous catalysts based on Cu(II) complexes designed for the synthesis of optically pure propargylamines were described. For the heterogeneous variant of enantioselective synthesis of propargylamines, only a version of a Cu(I) pybox complex anchored on magnetic nanoparticles (Fe_3_O_4_@SiO_2_) have been described so far [31].

### 2.5. Nitroaldolization Reaction

**Scheme 7:**
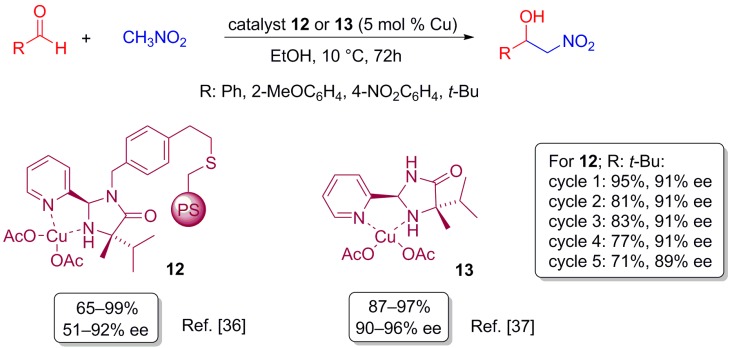
Henry reaction catalysed by copper(II) complex **13** and its immobilized form **12**.

The nitroaldolization (Henry) reaction catalysed by chiral optically pure catalysts is important among reactions; it involves the formation of a new carbon–carbon bond [32,33,34,35,36,37,38,39]. This reaction occurs in the synthesis of substituted 2-nitroalcohols used e.g., for the preparation of biologically active compounds and medicinal drugs [34]. A recently published comprehensive paper [35] covers works concerning catalysts based on supported transition metal complexes aimed for an asymmetric variant of the Henry reaction. Three recent works were not covered in the above mentioned review [36,38,39]. The first work [39] included a study of a library of substituted polystyrylsulfonyl-imidazoline-aminophenol copper complexes as solid supported catalysts for the Henry reaction. The selected complex of copper(II) acetate, based on (*S*,*S*)-diphenylethylenediamine-derived imidazoline, (*S*)-phenylethylamine, and dibromophenol, was highly efficient and provided the products of the reaction (nitromethane with substituted aldehydes) in high yields (76–99%) and high enantioselectivity (75–95% ee). The same ligand as a complex with copper(II) triflate was examined in the Friedel–Crafts alkylation of indole by substituted nitroalkenes, to give the adducts in high yields (86–99%) and high enantioselectivity—up to 83% ee [39]. The second variant of immobilization represented recyclable the heterogeneous catalyst **12** (Scheme 7), containing imidazolidine-4-one covalently bound on swellable pearl-like polystyrene having an –SH group (200–800 µm) [36]. Covalent anchoring of the ligand was performed by radical thiol-alkene click reaction, initiated thermally (azobisisobutyronitrile (AIBN), toluene, reflux 24 h), as well as photochemically (2,2-dimethoxy-2-phenylacetophenone, λ_max_ = 365 nm, 40 W, DCM, 25 °C, 24 h). For the subsequent reaction of the copolymer with copper(II) acetate, heterogeneous catalyst **12** was prepared, and was characterized by elemental microanalysis and Raman spectroscopy [36]. Henry reactions proceeded in the polymeric matrix of swellable catalyst **12** with a rate comparable with the use of catalyst **13** in homogeneous media [37]. The corresponding substituted (*R*)-2-nitroethanols were produced in high yields (65–99%) and with high enantioselectivity (up to 92% ee) comparable with the homogeneous catalyst **13** (up to 96% ee). The reuse of catalyst **12** was studied for the reaction of pivalaldehyde with nitromethane. After five-fold recovery and reuse of heterogeneous catalyst **12**, a decrease in yield of about 24% occurred; on the other hand, the decrease in enantioselectivity was negligible (3% ee) (Scheme 7) [36].

**Scheme 8:**
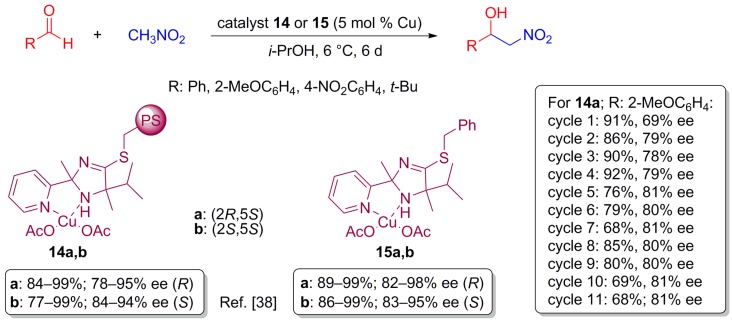
Henry reaction catalysed by copper(II) complexes **15a**,**b** and their immobilized forms **14a**,**b**.

The third variant represented heterogeneous catalysts **14a**,**b** based on Cu(II) complexes of substituted imidazolidine-4-thiones immobilized by means of sulfur atoms on different types of polystyrene carriers (polystyrene-*co*-4-vinylbenzyl chloride-*co*-tetra(ethylene glycol)-bis(4-vinylbenzyl)ether), Merrifield resin™ and JandaJel resin™ (Scheme 8) [38]. It was found, that catalysts **14a**,**b** catalyse the Henry reaction with high yields and high enantioselectivity, regardless of the polymeric carrier used (up to 98% ee). The obtained yields and enantioselectivities were comparable with the individual cases of reactions catalysed by analogical homogeneous catalysts **15a**,**b** (Scheme 8). Beside monitoring the yields and enantioselectivity, the possibility of recovery and reusing of catalysts **14a**,**b** was also studied. For instance, in the reaction of 2-methoxybenzaldehyde with nitromethane, the catalyst was reused more than 10 times without any decrease in enantioselectivity (81% ee) (Scheme 8) [38].

### 2.6. Asymmetric Friedel–Crafts Reaction

**Scheme 9:**
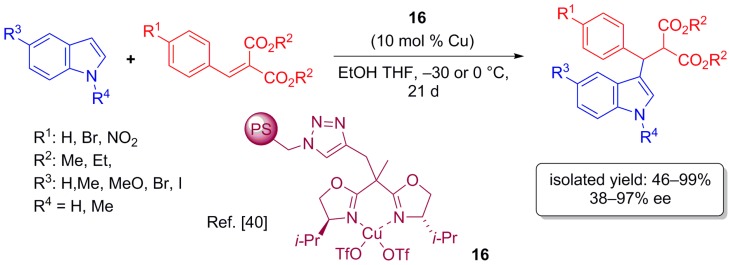
Asymmetric Friedel–Crafts alkylation of indole catalysed by polymeric complex **16**.

Beletskaya et al. [40] studied asymmetric Friedel–Crafts alkylation of indole and its derivatives in the presence of complex of Cu(OTf)_2_ with chiral isopropyl bis(oxazoline) ligand immobilized on Merrifield resin™ according to the “click” procedure **16**. The target products were obtained with a yield of up to 99% and up to 97% ee. Catalyst **16** ensured high yield and enantioselectivity even after five-fold reuse (Scheme 9) [40].

The heterogeneous catalyst **16** was also tested in the alkylation of *N*-methylindole by methyl (*E*)-2-oxo-4-phenylbut-3-enoate with yields of 22–94% and lower enantioselectivity (49% ee) [40].

### 2.7. Asymmetric Mukaiyama Aldol Reaction

**Scheme 10:**
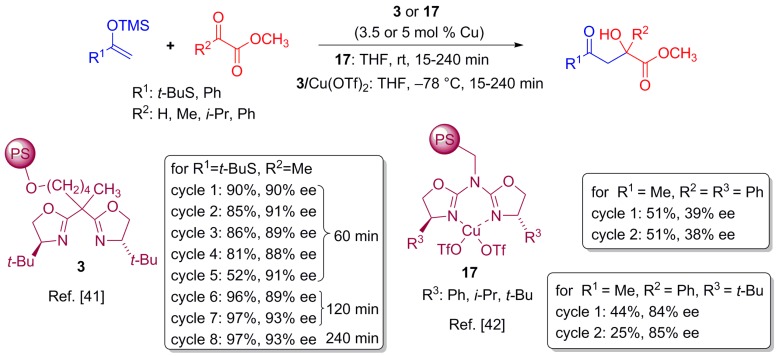
Asymmetric Mukaiyama aldol reactions catalysed by complexes of Cu(OTf)_2_/**3** and/or **17**.

The enantioselective Mukaiyama aldol reaction of methyl pyruvate with silylated ketene thioacetal was tested with a heterogeneous polystyrene copper complex catalyst based on chiral bis(oxazoline) [41] (Scheme 10).

The highly-crosslinked polymer **3** was prepared by a copolymerization strategy in the presence of toluene as a porogen agent [41]. The heterogeneous catalyst **3**/Cu(OTf)_2_ was less active than the analogous homogeneous catalysts, and aldol products were formed after a longer time; however, high overall yield (90%) and with an enantiomeric excess comparable to those obtained with the soluble ligands (90% ee), was obtained. Moreover, the catalyst based on polymer complex **3** was easily recovered by simple filtration and was reused several times without any loss of activity or stereoselectivity (Scheme 10) [41]. In the second work, the azabis(oxazoline) complexes **17** bonded to Merrifield resin™ were confirmed for the aldol reaction of different α-ketoesters with silylated ketone enolates [42]. The catalysis results showed that these heterogeneous complexes **17** accomplished enantioselectivities only slightly lower than those obtained in solution. Catalyst recovery depends on the ligand, so whereas polymers (R^3^: Ph) and (R^3^: *i*-Pr) were recovered with the same unsatisfactory results (51%, 38–39% ee), the recovered catalyst coming from (R^3^: *t*-Bu) led to even slightly lower yields (25%) but with the higher enantioselectivity (85% ee) (Scheme 10) [42].

## 3. Carbon–Nitrogen Forming Reactions

### 3.1. Copper(I)-Catalysed Azide Alkyne Cycloaddition

Beside the reactions leading to carbon–carbon bond formation, the reactions providing new carbon–nitrogen bond also play a relevant role in organic synthesis. Particularly, this includes the Huisgen 1,3-dipolar cycloaddition of alkynes with azides providing 1,4- and 1,5- isomers of 1,2,3-triazole [43]. Copper(I)-catalysed azide alkyne cycloaddition (CuAAC) represents a current variant, which converse to the original thermic version, proceeds quickly, regiospecifically (1,4-), and with high yields [44,45]. These properties rank this reaction among the most significant “near-perfect” bond-forming reactions known as “click” reactions [46]. The importance of this reaction consists not only in the efficient preparation of substituted 1,2,3-triazoles, but especially in the possibility for connecting different molecules and macromolecules, or to functionalize surfaces in a controlled way. The first polystyrene-supported copper(I) catalyst for CuAAC was based on the Amberlyst A21–CuI complex and was published in 2006 [47]. This and many further papers are discussed in a recently published review concerning immobilized copper complexes anchored on polystyrene and other heterogeneous carriers [48]. Also, two other papers deal with CuAAC [49,50].

**Scheme 11:**
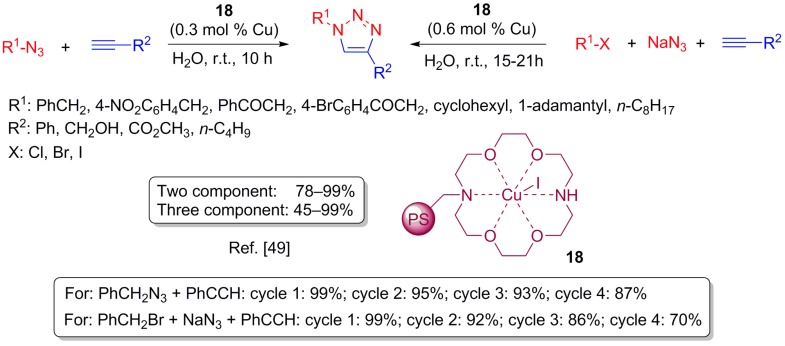
Huisgen 1,3-dipolar cycloaddition catalysed by polystyrene supported complex **18**.

**Scheme 12:**
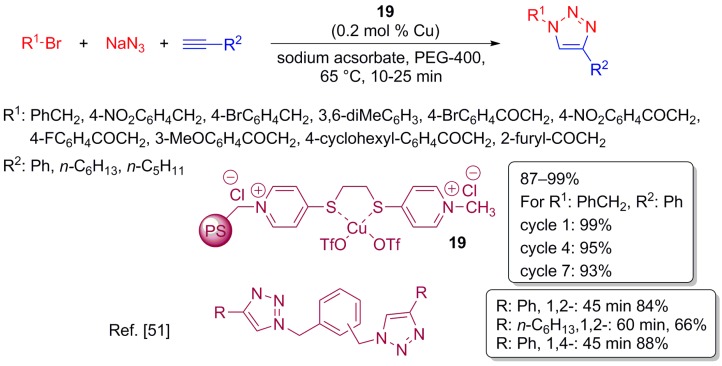
Huisgen 1,3-dipolar cycloaddition catalysed by polystyrene supported complex **19**.

The authors [49] prepared the catalyst **18** based on copper(I) iodide complex with cryptand-22 immobilized on commercial chloromethylated polystyrene (1% DVB) (Scheme 11). This heterogeneous heat- and air-stable catalyst **18** was used for a two-component CuAAC reaction of substituted organic azides with substituted alkynes, and this provided excellent yields of 1,4-disubstituted 1,2,3-triazoles (78–99%). The second three-component variant of this reaction involving organic halides, sodium azide and alkynes, also resulted in the production of 1,4-disubstituted 1,2,3-triazoles with good yields (45–99%). Both variants of the reaction were performed under eco-friendly conditions (0.6 mol % Cu, aerobic, water, room temperature). The reusing of catalyst **18** was successfully tested for the reaction of benzyl azide with phenylacetylene (Cycle 1: 99%; Cycle 4: 87%) and for the variant of the reaction of benzyl bromide, sodium azide and phenylacetylene (Cycle 1: 99%; Cycle 4: 70%) (Scheme 11) [49]. The catalyst **18** was also successfully used for the formation of aryl–sulphur bonds in aqueous media [50]. In the next work [51], the heterogeneous catalyst **19** based on a copper(II) complex of 1,2-bis(4-pyridylthio)ethane anchored on chloromethylated polystyrene was prepared (Scheme 12). Catalyst **19** was tested for three-component reaction of organic halides, sodium azide and alkynes, which was performed in the presence of sodium ascorbate as a reduction agent in poly(ethylene glycol) (PEG-400), (0.2 mol % Cu), at 65 °C, 10–25 min. The yields of isolated 1,4-disubstituted 1,2,3-triazoles were excellent, ranging from 87–99%. For the reaction of 4-bromobenzylbromide, sodium azide and phenylacetylene catalysed by **19** were further tested different solvents (0.1–0.3 mol % Cu, 15–30 min): water (78%), methanol (70%), toluene (40%), DMF (65%), MeCN (28%), PEG–water 1:1 (89%), ethanol–water 1:1 (75%).

The recovery and reuse of catalyst **19** was successfully confirmed with the same three-component reaction (Cycle 1: 99%; Cycle 4: 95%; Cycle 7: 93%) (Scheme 12). This catalytic system also showed excellent activity for the synthesis of *bis*-1,4-disubstituted 1,2,3-triazoles (66–88%) [51].

### 3.2. Other Ring-Formation Reactions

**Scheme 13:**
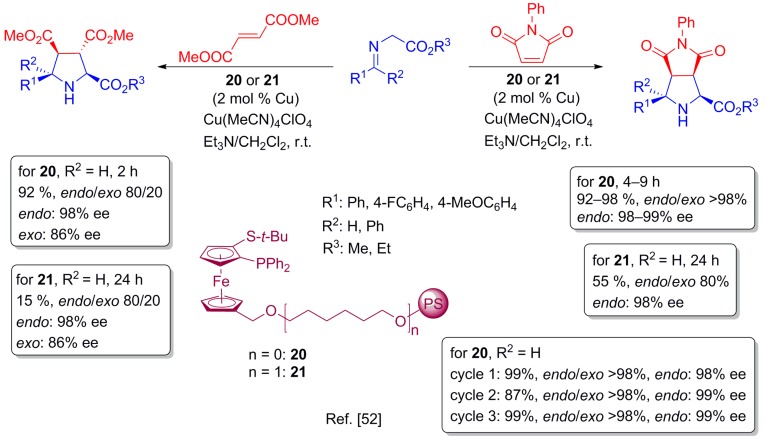
Asymmetric 1,3-dipolar cycloadditions catalysed by polymeric complexes of Cu(MeCN)_4_ClO_4_ with **20** and or **21**.

Carretero et al. [52] studied the Cu(I)-catalysed asymmetric 1,3-dipolar cycloaddition of azomethine ylides generated from substituted arylimines derived from glycine esters with *N*-phenylmaleimide or dimethyl fumarate (Scheme 13). Except for the homogeneous variant, cycloaddition was studied in the heterogeneous system with the use of in situ prepared complexes Cu(MeCN)_4_ClO_4_ with Fesulfos-based chiral ligands immobilized on Merrifield′s peptide resin (1% cross-linked, 200–400 mesh, 2 mmol Cl/g) **20** or on trichloroacetimidate Wang resin (copoly(styrene-1% DVB), 200–400 mesh, 0.6–1.00 mmol trichloroacetimidate/g resin) **21**.

Interestingly, **20** showed much better catalytic performance than **21** (in all cases from 91 to >99% ee). Furthermore, the polystyrene-supported Fesulphos ligand **20** were recovered and reused in successive catalytic 1,3-dipolar cycloadditions without further addition of Cu(I), maintaining excellent enantioselectivity in up to three runs (Scheme 13) [52].

Halligudi et al. studied biomimetic oxidative coupling of 2-aminophenol to phenoxazine-2-one catalysed by bis(2′-[2-hydroxyethyl]benzimidazolato)copper(II) complex anchored onto chloromethylated polystyrene [53]. The kinetics and the effects of the catalyst concentration, substrate concentration, air pressure and temperature on the reaction course were studied. 2-Aminophenol in the presence of air (70 °C, 800 psig, DMF, 8 h) gave 62% conversion and the immobilized catalyst was more stable and more active than its homogeneous analogue. The catalyst recycling study demonstrated that the anchored catalyst did not leach out the metal complex during the reaction, and thus its catalytic activity was reproducible [53].

### 3.3. N-Arylation of Amines, Amides and Nitrogen Containing Heterocycles

**Scheme 14:**
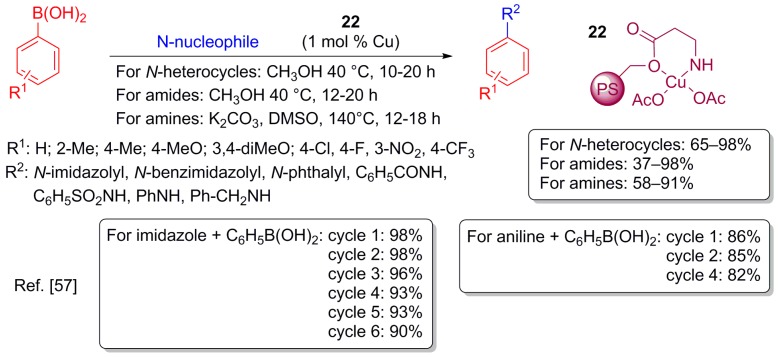
*N*-Arylation of *N*-nucleophiles by substituted phenylboronic acids catalysed by polystyrene supported complex **22**.

Aryl–nitrogen bonds formed via cross-coupling reactions represent a powerful tool for the production of a number of compounds with practical uses [54]. The original variant of the Ullmann reaction is still utilized for this purpose [55]. However, this method of performing of the reaction is constrained by several disadvantages, e.g., it needs high temperature (150–200 °C) and the use of stoichiometric amounts of copper [54,55]. A remarkable simplification was achieved by the use of substituted phenylboronic acids for *N*-arylation of imidazole catalysed by copper(II) salts [56]. The next facilitation was the utilization of heterogeneous catalyst **22** (Scheme 14) [57]. Catalyst **22** was prepared from commercial chloromethylated polystyrene (5.5% DVB), on which β-alanine was attached through the oxygen atom, and copper(II) acetate was coordinated. Less efficient variants of this catalyst were prepared by the coordination with CuCl_2_ and CuI. Catalyst **22** was successfully tested for the reaction of substituted phenylboronic acids with nitrogen containing heterocycles (imidazole, benzimidazole; 65–98%) and with amides (phthalimide, benzamide, benzenesulfonamide; 37–98%) under very mild conditions (methanol, 40 °C, 10–20 h). The reaction with amines was performed in DMSO in the presence of K_2_CO_3_ at 140 °C (aniline, benzylamine; 58–91%) (Scheme 14) [57].

The recovery and reuse of catalyst **22** was studied for the reaction of phenylboronic acid with imidazole (Cycle 1: 98%; Cycle 6: 90%) and aniline (Cycle 1: 86%; Cycle 4: 82%). In both cases, only a slight decrease in yields was observed (Scheme 14) [57]. Under comparable conditions as in [57] (catalyst **22**), the same authors achieved analogical *N*-arylations of N(H)-heterocycles with aryl halogenides and arylboronic acids with good or excellent results with the use of heterogeneous catalysts based on complexes of polystyrene–Schiff bases (PS-4-C_6_H_4_N=CH-2-pyridyl Cu(OAc)_2_) [58], or (PS-4-C_6_H_4_N=CH-2-furyl Cu(OAc)_2_) [59].

**Scheme 15:**
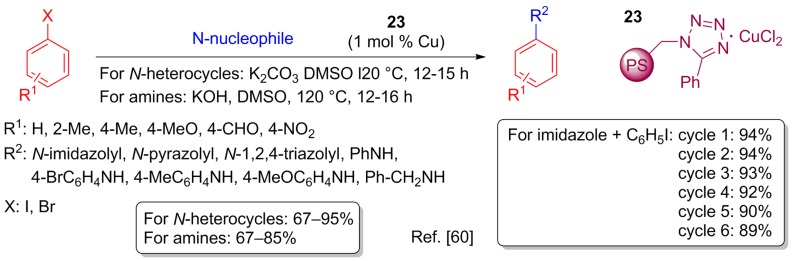
*N*-Arylation of *N*-nucleophiles by substituted aryl halides catalysed by polystyrene supported complex **23**.

Another variant of this reaction is the *N*-arylation of imidazole, pyrazole, 1,2,4-triazole, benzylamine and substituted anilines with substituted iodobenzenes catalysed by catalyst **23** (Scheme 15) [60].

**Scheme 16:**
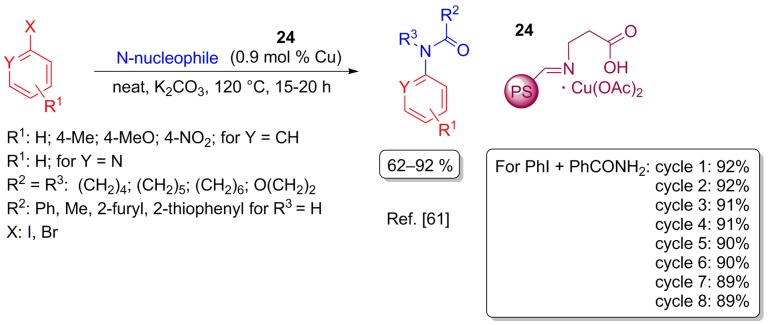
*N*-Arylation of *N*-nucleophiles by substituted aryl halides catalysed by polystyrene supported complex **24**.

Catalyst **23** was prepared from commercial chloromethylated polystyrene (2% DVB), on which 5-phenyl-1*H*-tetrazole was attached, and CuCl_2_ was coordinated (Scheme 15). The reactions were performed in DMSO at 120 °C for 12–16 h in the presence of K_2_CO_3_ (*N*-heterocycles; 67–95%), or in the presence of KOH (amines; 67–85%). In different solvents (THF, MeCN, DME, *N-*methylpyrrolidone (NMP), DMF) and under different conditions (Cs_2_CO_3_, NaHCO_3_, K_3_PO_4_, TEA; 80 °C, 130 °C) *N*-arylation of 1*H*-imidazole with iodobenzene occurred with lower yields than the reaction in DMSO. The recovery and reusing of catalyst **23** was tested with success for the reaction of imidazole with iodobenzene (Cycle 1: 94%; Cycle 6: 89%). The *N*-arylation of amides and lactams with substituted iodobenzenes, bromobenzene, 2-bromopyridine or 2-iodopyridine catalysed by **24** was also successful (Scheme 16) [61].

**Scheme 17:**
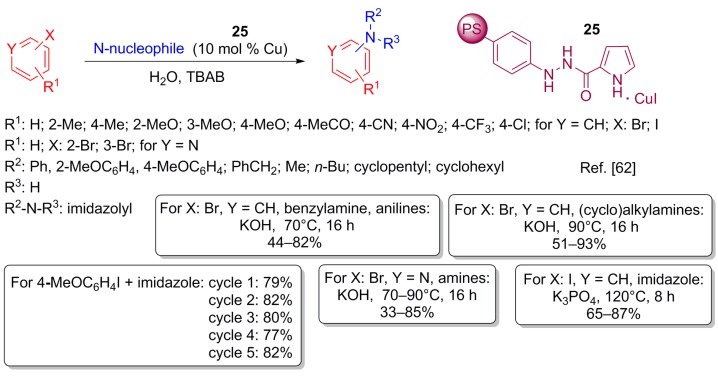
*N*-Arylation of *N-*nucleophiles by substituted aryl halides catalysed by polystyrene supported complex **25**.

Catalyst **24** was prepared from commercial chloromethylated polystyrene, where the chloromethyl group was transformed to an aldehydic group. Subsequent reaction with β-alanine led to the production of an imine, which was then coordinated with Cu(II) acetate. *N*-arylations were performed without a solvent and in the presence of K_2_CO_3_ at 120 °C, for 15–20 h, with yields in the range of 62–92%. The recovery and reusing of catalyst **24** was successfully tested for the reaction of benzamide with iodobenzene (Cycle 1: 92%; Cycle 8: 89%) (Scheme 16) [61]. Further examples of reactions proceeding in aqueous media were *N*-arylation of substituted bromobenzenes with substituted anilines and (cyclo)alkylamines, catalysed by catalyst **25** in the presence of tetrabutylammonium bromide (TBAB) (Scheme 17) [62]. Catalyst **25** was prepared by the sequence of reactions from atactic polystyrene. Firstly, polystyrene was nitrated, reduced to amine, diazotated, and reduced to hydrazine, which was then acylated by 1*H*-pyrrole-2-carboxylic acid. The catalyst **25** itself was generated directly in the reaction mixture from the functionalized polystyrene and Cu(I) iodide.

The reaction of substituted bromobenzenes with benzylamine and substituted anilines (70 °C, 16 h) proceeded with very good yields of up to 82%. The lowest yield was attained in the reaction of 4-bromonitrobenzene with benzylamine (44%), while the reaction with sterically demanding 2,4,6-trimethylaniline did not proceed. Analogical reactions with (cyclo)alkylamines (90 °C, 16 h) provided yields of up to 93%, while the lowest yield was achieved in the reaction of 4-bromonitrobenzene with butylamine (51%). Substituted bromopyridines provided products with yields of 33–85%. The reactions of substituted iodobenzenes with imidazole (120 °C, 16 h) led to products with yields ranging from 65% to 87%. The lowest yield was obtained for iodobenzene itself (65%), while the reaction with 2-methoxyiodobenzene did not proceed. Catalyst **25** was also applied in the intramolecular *N*-arylation of *N*-(2-iodophenyl)-1*H*-imidazole-2-carboxamide giving imidazo[1,2-a]quinoxaline. The recovery and reusing of catalyst **25** was successfully tested for the reaction of 4-methoxyiodobenzene with imidazole (Cycle 1: 79%; Cycle 5: 82%) (Scheme 17) [62].

### 3.4. Aza-Michael Addition

**Scheme 18:**
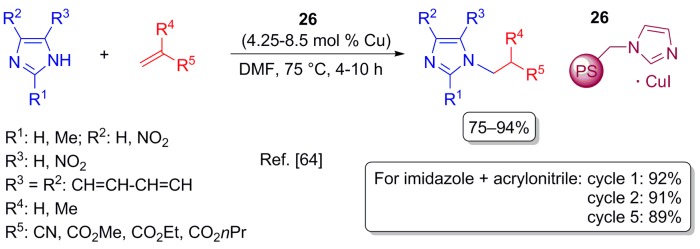
Aza-Michael addition catalysed by polystyrene supported complex **26**.

Nucleophilic addition of *N*-nucleophiles on activated multiple bonds (aza-Michael addition) is a significant reaction in the synthesis of β-amino carbonyl compounds as crucial intermediates in the synthesis of β-amino acids, β-lactam antibiotics, and chiral auxiliaries [63]. Simple, green and efficient preparation of *N*-alkylated imidazoles was tested, with a reaction of substituted imidazoles with different α,β-unsaturated compounds catalysed by polystyrene-supported CuI–imidazole complex catalyst **26** (Scheme 18) [64]. Catalyst **26** was prepared via surfactant free emulsion polymerization with 4-VBC (4-vinylbenzylchloride) and DVB (2%), whereby uniform globular particles (400–500 nm) with high Cl content (21.5%) were obtained initially. Afterwards, these particles were highly functionalized by imidazole and coordinated by CuI.

The efficiency of catalyst **26** was firstly confirmed for the addition of imidazole on acrylonitrile (8.5 mol % Cu; 60 °C; 4–10 h); while the influence of the solvent was studied: methanol (58%); MeCN (63%); DMSO (52%); DMF (71%); acetone (47%); THF (58%). The reactions were then performed in DMF and the temperature optimized to 75 °C. The addition of substituted imidazoles on α,β-unsaturated substrates proceeded with very good yields up to 74–84%. The lowest yield was achieved for the addition of 4-nitro-1*H*-imidazole on acrylonitrile. Catalyst **26** was separated by filtration and was further recycled for the reaction of imidazole with acrylonitrile (Cycle 1: 92%; Cycle 2: 91%; Cycle 5: 89%). The given results show that catalyst **26** represents an excellent reusable catalyst; which could be potentially applied at an industrial scale [64].

## 4. Conclusions

In this mini-review, we focused on Cu(I) and Cu(II) complexes anchored on pearl-like polystyrenes. The prepared heterogeneous catalysts were beneficially used for oxidative homocoupling of terminal alkynes, synthesis of propargylamines, nitroaldolization reaction, azide alkyne cycloaddition, *N*-arylation of nitrogen containing compounds, aza-Michael addition asymmetric Friedel–Crafts reactions, asymmetric Mukaiyama aldol reactions, and asymmetric 1,3-dipolar cycloaddition of azomethine ylides. In several given examples, any difference between the efficiency of individual catalysts was not found depending on the method of their preparation (copolymerization strategy or post-modification strategy) [15,16,17,18,19]. In a prevalent number of examples, any significant decrease in chemical yields was not observed in the comparison of heterogeneous catalysts with corresponding homogeneous catalysts. The advantages of discussed heterogeneous catalysts consisted in their simple preparation from inexpensive and easily available starting materials, their simple isolation, and the possibility of reuse. Easy performance of the reaction in aqueous media brings great benefits in the case of cross-coupling reactions [13], Huisgen cycloaddition [49], *N*-arylation [62], and solvent-free *N*-arylation [61]. Most of the mentioned reactions possess high potential for industrial usage, hence the catalytic systems presented in this review significantly contribute to further development of ecologically sustainable chemical processes and technologies.
